# Ecological and functional adaptations to water management in a semiarid agroecosystem: a soil metaproteomics approach

**DOI:** 10.1038/s41598-017-09973-w

**Published:** 2017-08-31

**Authors:** Robert Starke, Felipe Bastida, Joaquín Abadía, Carlos García, Emilio Nicolás, Nico Jehmlich

**Affiliations:** 1 Helmholtz-Centre for Environmental Research – UFZ, Department of Molecular Systems Biology, Permoserstrasse 15, 04318 Leipzig, Germany; 2 Centro de Edafología y Biología Aplicada del Segura. Spanish Research Council (CEBAS-CSIC). Campus Universitario de Espinardo, CP 30100 PO Box 164, Murcia, Spain

## Abstract

Climate change models point to a decrease in water availability in semiarid areas that would compromise the maintenance of sustainable agriculture. Here, we used a grapefruit agroecosystem model to evaluate the responses of the active soil microbial community – as a microbial subset directly involved in soil functionality- undergoing strategies to cope with the low water availability in south-east Spain. For this purpose, we tested the impacts of: (i) water quality: transfer-water from a river (TW) or reclaimed-water from a wastewater-treatment plant (RW); and (ii) water quantity: continuous optimal amount of water or reduced irrigation (RDI) in the temporal frame when the crop is less sensitive; and their interactions. Metaproteomics revealed that the phylogenetic diversity of the active community and its functional diversity were lowered in soils with RW. RDI lowered soil respiration and functional diversity while the phylogenetic diversity remained constant. The reestablishment of full irrigation after RDI led to a recovery of soil respiration that was accompanied by an enhanced abundance of resilient bacterial populations. Bacterial populations displayed molecular mechanisms against water stress that have been conserved evolutionarily in plants. Protein-based studies shed light on ecological and functional mechanisms that govern the adaptive responses of soil microbial communities to climate-change friendly water management.

## Introduction

Climate change will impact natural habitats through decreasing precipitation accompanied by a rise in temperature and evapotranspiration in many arid and semiarid ecosystems^1^. The deficit of available water is especially fateful in semiarid areas, such as south-east Spain^2^, that strongly rely on sustainable agriculture as an economic resource. In these conditions, alternative water management regimes, that aim to maintain the sustainability of agroecosystems in highly productive areas, should be investigated.

At the level of plant physiology and productivity, the reduction of the volume of irrigation water in summer when some fruit species are less sensitive - which is referred to as regulated deficit irrigation (RDI) - seems to be an alternative strategy to cope with water limitation in semiarid agroecosystems without altering the wellness of *Citrus* sp. crops. Another strategy to tackle water limitation could be the use of reclaimed water from wastewater treatment plants. This water constitutes a continuous and cheap resource for agriculture when water demands are not satisfied by the availability of natural sources of water^3, 4^.

Several studies have highlighted that irrigation with reclaimed water does not negatively impact on the productivity and physiology of grapefruits and mandarins^3, 4^. However, its consequences for the soil microbial community are less known^5^. The determination of the latter is of great interest as soil microbial communities play a key role in soil fertility by driving organic matter (OM) cycling and nutrient generation for plants. However, the utilization of reclaimed water is not exempt from risks. In fact, high amounts of salts in reclaimed water may lead to soil salinization, which then decreases microbial biomass and enzymatic activity^6^. Furthermore, reclaimed water may have some risks related to the potential transfer of pathogens to food-webs^7^. Conversely, the large amounts of soluble OM contained in reclaimed water can benefit microbial growth and enzyme activities and alter the composition of the microbial community^7, 8^.

The impact of soil moisture and water availability on the performance of soil microbial communities in natural and forest ecosystems has been studied at the levels of microbial growth^9–11^, microbial community composition, and biogeochemical cycles. However, the activity of soil microbial populations is not easily tracked. For instance, many cells can be in dormant status in soil which limits their activity. Moreover, high content of “relic DNA” belonging to dead cells –that can count up to 40% of the soil DNA^12^ - obscures the connections between environmental factors and the real activity of microbial communities. Particularly, it is important to highlight that the active fraction of the community has been proposed to be more reactive to environmental variations in soil than the total community analyzed by bacterial (i.e. 16S rRNA gene) and fungal (i.e. ITS) genomic profiling^13^. Moreover, metatranscriptomics tracks the active microbial populations by following the expression of genes to mRNA^14^. Indeed, metatranscriptomics was recently used to investigate the activity of microbial communities in soil^15–17^ but metaproteomics can help to link the active populations to their actual functionalities. The active microbial community is a subset of the total community which is responsible for the functional processes in soil (i.e. OM mineralization, soil fertility, nutrient cycling, etc.) and its responses can often be masked by the total community^18^. Most methods partially fail to evaluate the active fraction of microbial communities, which represents only 0.1–2% of the total biomass in soil^19^. As a consequence, the impacts of agricultural practices can be misinterpreted if the active microbial community is not tackled properly. Considering the active community would change our perception of microbial-mediated ecosystem processes and our capacity for modeling the impacts of climate change and adaptations to it.

The development and accuracy of new mass spectrometers coupled to nano-HPLC systems, together with improvement of extraction methods and implementation of genomic databases, have fostered soil metaproteomics studies^20, 21^ and have provided unprecedented insights into soil microbial ecology^22, 23^. Tracking the protein repertoire in environmental samples (so-called metaproteomics) enables the identification of the active microbial populations and their cellular functionalities^20, 23, 24^. Furthermore, metaproteomics can help to understand attributes of ecological relevance such as the resistance and resilience of soil microbial communities against water management^25^. The resistance of the active soil microbial community to disturbance and the capacity to return to the original or another, alternate state after disturbance (so called resilience) are components of ecosystem stability^26, 27^. Here, we evaluate the resistance of the active soil microbial community to reduced irrigation and its resilience once the normal application of irrigation water is re-established in a Mediterranean grapefruit orchard. The resistance and resilience will be evaluated also in relation to the interaction with water quality. For this purpose, the impacts of water of high quality transferred from a river channel and reclaimed water from a wastewater treatment plant will be assayed. Reclaimed-water from wastewater treatment plants has elevated salt and soluble OM contents. Consequently, the repeated irrigation with this water could cause chemical changes in soil that include the increase of electrical conductivity (EC) and further impacts on microbial biomass and community composition^5, 8^. However, knowledge of the activity of soil microbial populations in relation to water management such as reduced irrigation or the irrigation with reclaimed water is still scarce.

We suggest the following hypothesis. Firstly, reduced irrigation will enhance the activity of drought-tolerant bacterial populations such as *Actinobacteria* and *Firmicutes*. Conversely, reduced irrigation will reduce the activity of populations that usually correlate to soil moisture, i.e. *Proteobacteria* ^18, 28^. Since *Proteobacteria* is a highly-diverse phylum, reduced irrigation is expected to drop the active phylogenetic diversity, as estimated by the Shannon-Wiener index. Secondly, considering the immense microbial diversity in soil^29^ and its plasticity^30^, we expect that the active phylogenetic diversity and the functional diversity will recover (resilience) once the optimum water quantity is resupplied after the RDI event. Thirdly, the resilience of soil respiration and microbial diversity will be mediated by water quality. The irrigation with reclaimed water will provide soluble organic matter to microbes and benefit the activity of copiotrophs such as *Proteobacteria* populations^22^, but at the same time it would select specific populations which are able to be active and survive under high salinity.

## Results

### Water characterization

Significant differences existed between the reclaimed water (RW) and the water transferred from the channel (TW) (Table 1). The RW showed a lower pH and higher salinity and sodicity than TW, which had lower values of EC (1.00 dS m^−1^) and a sodium absorption ratio of 1.39 [meq/l]^0.5^. In addition, RW had higher concentrations of NO_3_^−^, PO_4_^3−^, SO_4_^2−^, Cl^−^, Ca, Mg, K, B, and Na, compared to TW (Table 1). The concentrations of organic C and N in RW were 18 and 7 mg l^−1^, respectively.

**Table 1:** Physical and chemical analyses of water.

Parameter	Unit	TW	RW
EC_w_	dS m^−1^	1.00 ± 0.01	3.21 ± 0.20
pH		8.41 ± 0.09	7.70 ± 0.10
Ca	meq L^−1^	1.99 ± 0.10	3.58 ± 0.20
Mg	meq L^−1^	1.58 ± 0.10	3.92 ± 0.30
K	meq L^−1^	3.65 ± 1.40	38.94 ± 1.40
Na	meq L^−1^	1.86 ± 0.20	18.30 ± 1.20
B	meq L^−1^	0.01 ± 0.001	0.06 ± 0.004
Cl^-^	meq L^−1^	3.15 ± 0.40	20.10 ± 3.01
NO_3_^−^	meq L^−1^	0.38 ± 0.035	0.41 ± 0.081
PO_4_^3−^	meq L^−1^	0.01 ± 0.002	0.06 ± 0.007
SO_4_^2−^	meq L^−1^	5.90 ± 0.50	17.20 ± 3.41

Electrical conductivity (Ec_w_), pH, cations (Na, K, Ca, Mg), B, and anions (Cl^−^, NO_3_^−^, PO_4_^3−^, SO_4_^2−^) in 2015, for transfer water (TW) and reclaimed water (RW). Values correspond to annual averages for 2014 and 2015 ± standard deviation, with n = 12.

### Soil chemistry and respiration

As retrieved from the three-way ANOVA, the concentration of total organic C in the soil was affected by water quality but not by water restriction. In August, the soils irrigated with TW (TW-C and TW-RDI) had higher values of total organic C (16 and 15 g kg^−1^, respectively) than the soils irrigated with RW (RW-C and RW-RDI) (7.0 and 7.6 g kg^−1^, respectively). In August, the total N concentration was higher in TW-C (2.0 g kg^−1^) than in TW-RDI (1.4 g kg^−1^) and was higher in RW-RDI (1.5 g kg^−1^) than in RW-C (0.8 g kg^−1^).

**Figure 1:**
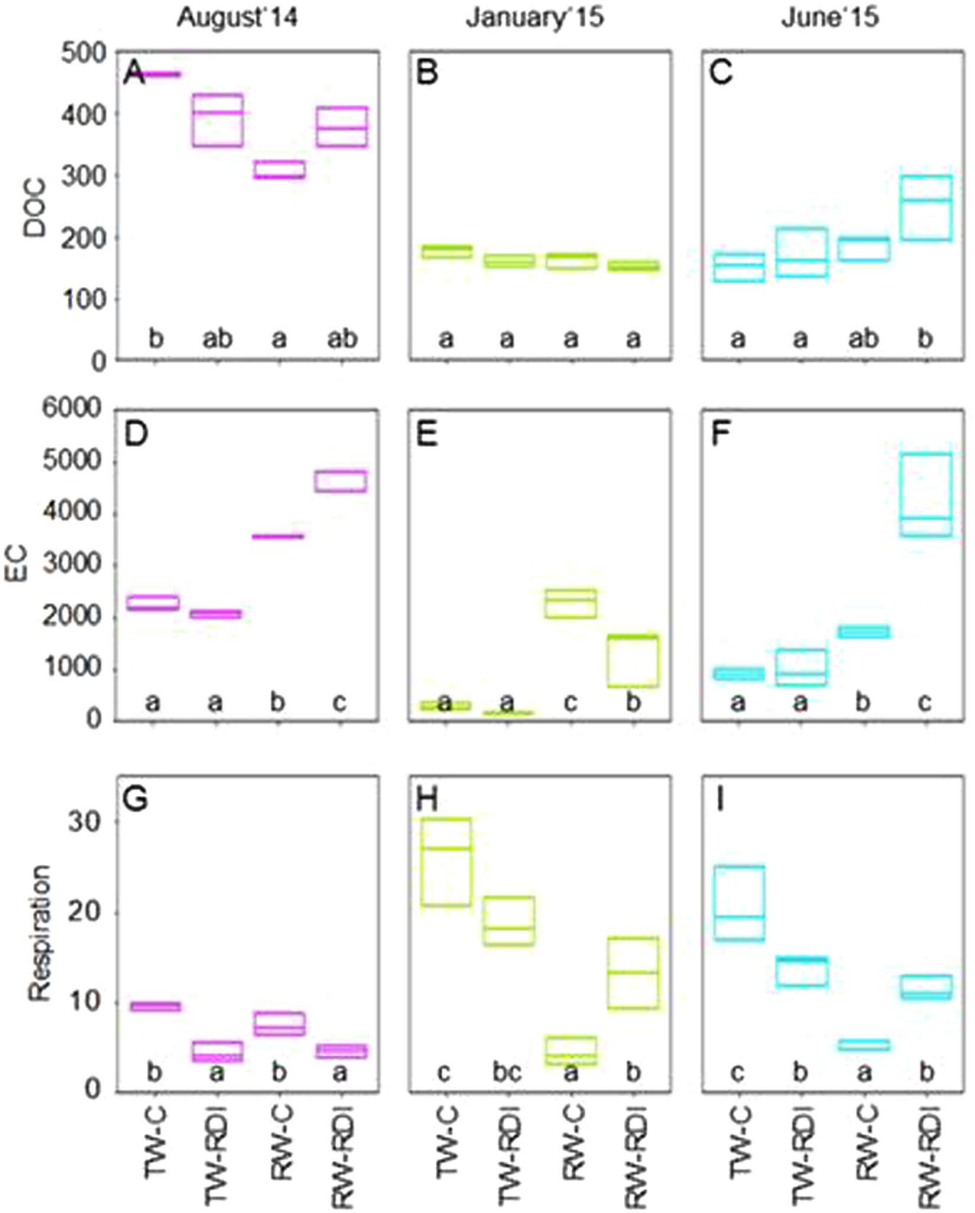
Time-dependent soil characteristics of the grapefruit field area. Dissolved organic carbon (DOC) (**A**–**C**), electric conductivity (EC) (**D**–**F**), and respiration (**G**–**I**) are shown. TW-C stands for transfer water control from Tajo River, RW-C for reclaimed water control and RDI for limited amount in summer (regulated deficit irrigation). The unit for respiration is mg CO_2_ per kg per day, for DOC mg C per kg and for EC µS per cm. Median, lower and upper quartiles are given. At each sampling time, data followed by the same letter are not significantly different according to the HSD test (*P* < 0.05).

DOC concentration was influenced significantly by sampling time (*P* < 0.05), but not by water quality or quantity (Fig. 1A and Table 2). Soil EC was influenced significantly by sampling time, water quantity, and water quality (*P* < 0.05) (Fig. 1B and Table 2). EC was always higher in soils irrigated with RW than in soils irrigated with TW. Soil respiration was influenced significantly by sampling time and water quality (*P* < 0.05), but not by water quantity (Fig. 1C and Table 2), as retrieved from the three-way ANOVA. It was generally higher in soils irrigated with TW than in soils irrigated with RW. The EC and soil respiration were negatively correlated (*P* < 0.0001; r = −0.65).

**Table 2:** Three-way ANOVA of single variables and PERMANOVA of the structure of microbial communities based on phylogeny and functionality of proteins.

	DOC		EC		Respiration		Active Diversity		Active Functionality	
	F	*P*	F	*P*	F	*P*	F	*P*	F	*P*
Quantity	1.246	0.275	12.41	0.002	0.58	0.015	0.1	0.75	8.83	0.007
Time	250.02	*	124.92	*	29.03	*	5.82	0.009	22.75	*
Quality x Quantity	14.02	0.001	17.47	*	26.58	*	1.44	0.24	0.16	0.69
Quality x Time	21.93	*	1.48	0.25	12.56	*	6.14	0.007	0.16	0.85
Quantity x Time	3.7	0.04	24.03	*	1.27	0.3	5.05	0.015	0.66	0.52
Quality x Quantity x Time	5.22	0.013	19.06	*	7.38	0.003	1.92	0.17	0.91	0.42
	**PCA Phylogeny**		**PCA Functionality**							
	**F**	***P***	**F**	***P***						
Quantity	2.88	0.0894	24.67	*						
Time	27.48	*	43.84	*						
Quality x Quantity	0.16	0.8	0.46	0.64						
Quality x Time	22.04	*	32.41	*						
Quantity x Time	7.52	0.0012	6.85	0.002						
Quality x Quantity x Time	20.72	*	12.81	*						

DOC (Dissolved organic carbon); EC (electrical conductivity); F (F ratio); P (P value); PCA (Principal component analysis); *P < 0.0001.

### The structure of the microbial community

**Figure 2:**
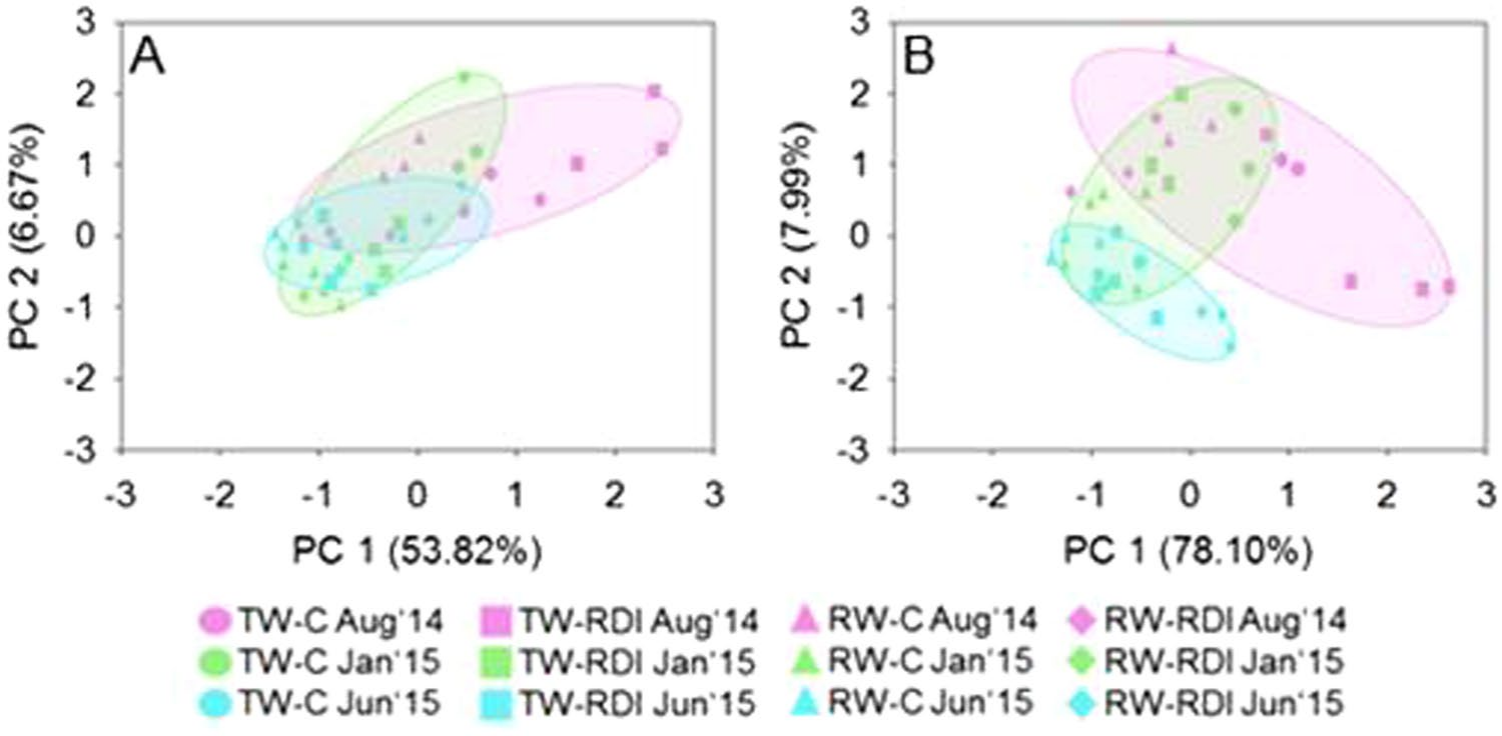
Principal component analysis with the relative abundances of proteins at the phylogenetic (**A**) and functional (**B**) levels.

The structure of the microbial community was subjected to PCA of the relative abundances of the orders analyzed by metaproteomics. The PCA of the phylogenetic data showed that the two first principal components (PC1 and PC2) accounted for more than 60% of the total system variance. Factor 1 explained 54% of the total system variance and factor 2 explained 7% (Fig. 2A). The following populations received a high absolute score in PC1: *Burkholderiales*, *Caulobacterales*, *Flavobacteriales*, *Jiangellales*, *Micrococcales*, *Myxococcales*, *Nitrosomonadales*, *Planctomycetales*, *Propionibacteriales*, *Rhodospirillales*, *Solirubrobacterales*, *Sphingobacteriales*, *Sphingomonadales* and *Xanthomonadales*. *Nevskiales* and *Thiotrichales* received a high loading score in PC2. PERMANOVA revealed a significant effect of water quality and sampling time, but not water quantity, on the structure of the microbial community (Table 2). Similarly, the interaction between water quality and sampling time and between water quantity and sampling time, and the triple interaction, influenced significantly the structure of the microbial community. Soil samples corresponding to the irrigation with RW (with or without RDI) were preferentially located on the negative side of factor 1. The structure of the microbial community in TW-RDI soil samples taken in August was clearly distinct from the rest.

At the functional level, PCA revealed changes in the functional structure of the microbial community. Factor 1 explained 78% of the total system variance while factor 2 explained 8% (Fig. 2B). Water quality and quantity and sampling time, as well as the interactions (except quality x quantity), influenced the functional structure of the microbial community (Table 2). Interestingly, again, samples from the RW treatments (with or without RDI) were mainly located on the left side of Factor 1 and samples of TW-RDI soil taken in August were clearly separated from the rest.

### Phylogenetic and functional diversity

**Figure 3:**
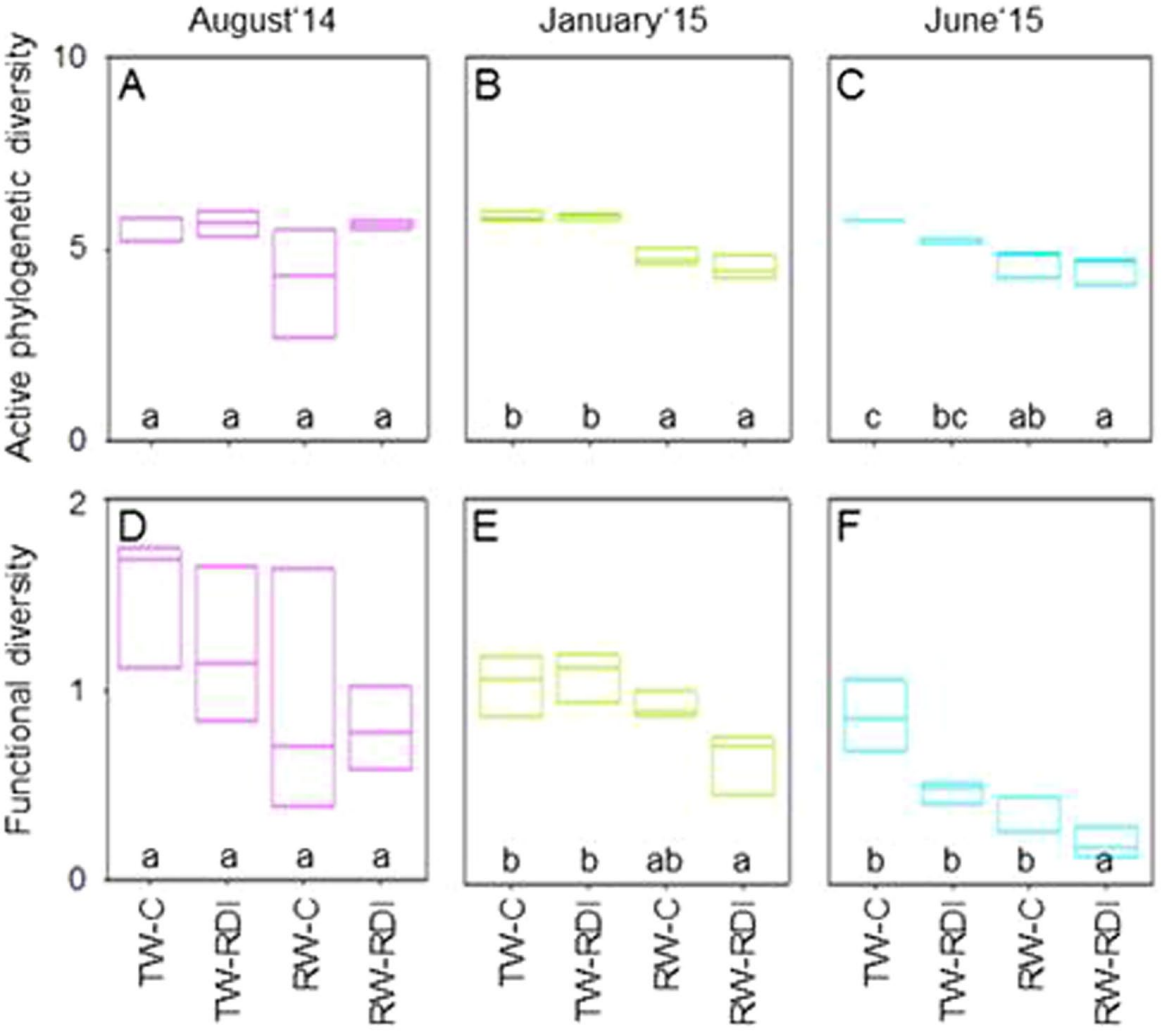
Time-dependent active phylogenetic diversity and functional diversity. TW-C stands for transfer water control river, RW-C for reclaimed water control and RDI for limited amount in summer (regulated deficit irrigation). Median, lower and upper quartiles are given. At each sampling time, data followed by the same letter are not significantly different according to the HSD test (*P* < 0.05).

Shannon-Wiener indexes were calculated using the relative abundance of proteins from microbial populations as a proxy of the active phylogenetic diversity (Fig. 3A–C) and using the cluster of orthologues groups (COGs) as an indicator of functional diversity (Fig. 3D–F). The active phylogenetic diversity was influenced significantly by water quality and sampling time, but not by water quantity (Table 2). The water quality x time and water quantity x time interactions, and the triple interaction, influenced the active diversity (Table 2).

Overall, the active phylogenetic diversity was higher in soils irrigated with TW than in those irrigated with RW (Fig. 3). The active phylogenetic diversity remained at the same level for TW-C over the course of the study, whereas TW-RDI led to high active diversity in August and January although it dropped slightly in June. For RW-C, the active diversity was 10% lower compared to the other treatments in August and remained at the same level over time. The active diversity of TW-RDI was similar to that of TW-C in August and January, but was lower in June. Treatment RW-RDI gave lower values than TW-RDI in January and June.

The values of functional diversity varied as follows: TW-C > TW-RDI > RW-C > RW-RDI, in August, January and June. The functional diversity was significantly influenced by water quality and quantity and sampling time (Table 2).

### The taxonomic composition of the microbial community

**Figure 4:**
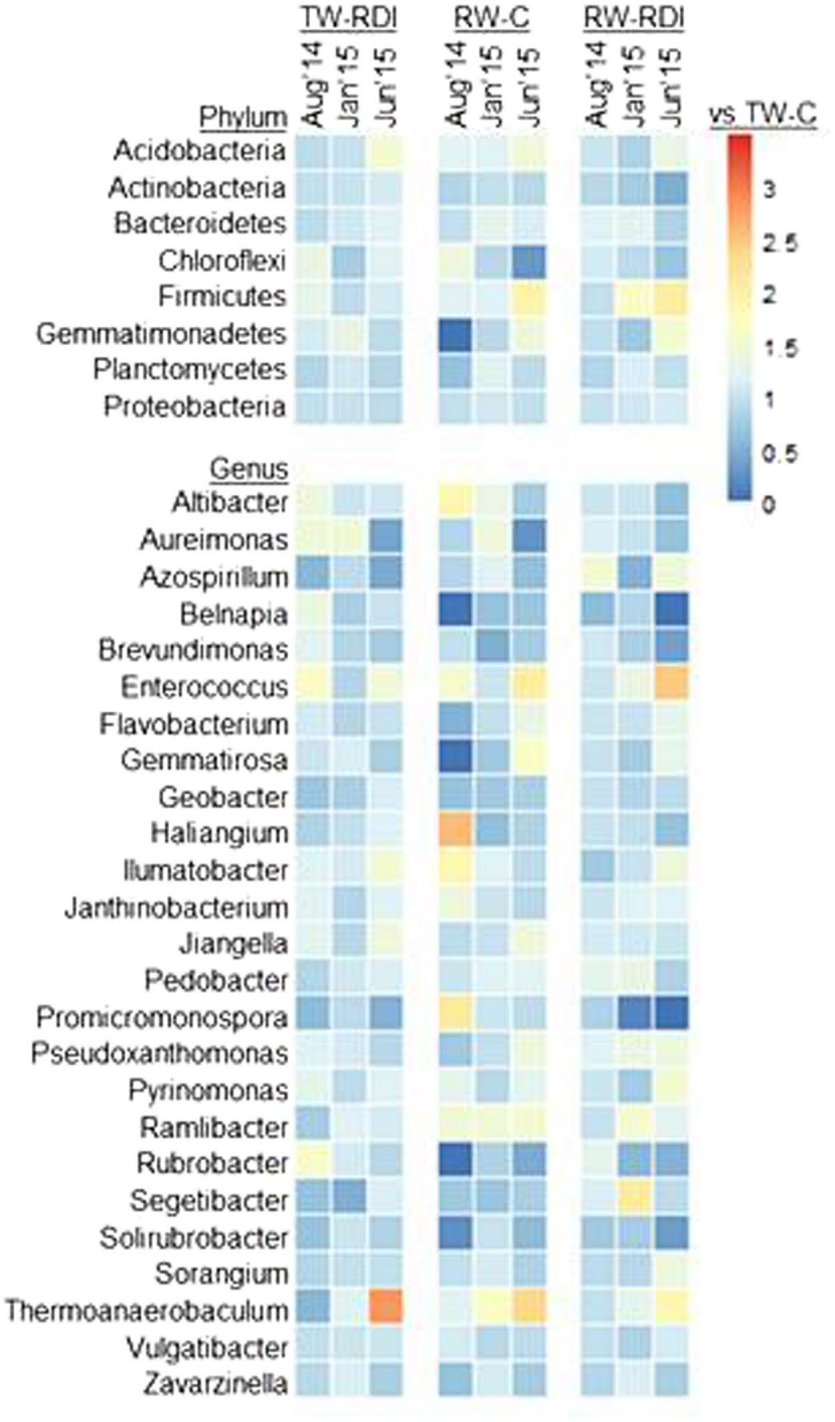
Change in relative abundances compared to transfer-water in optimal amounts (TW-C) per treatment and time at the level of phyla and the top25 genera. The relative abundance of each treatment and season was divided by the relative abundance of the control (TW-C) of the same treatment and season. TW-C stands for transfer water control from the Tajo river, RW-C for reclaimed water control, and RDI for limited amount in summer (regulated deficit irrigation).

The soil microbial community was dominated by *Proteobacteria* (up to 43.1% of the identified proteins), followed by *Actinobacteria* (up to 13.3%), *Bacteroidetes* (up to 9.5%), *Planctomycetes* (up to 6.9%), *Acidobacteria* (up to 5.2%), *Firmicutes* (up to 3.3%), *Gemmatimonadetes* (up to 2.4%), and *Chloroflexi* (up to 1.6%) (Supporting Information, Figure S1). Of the genera, *Pseudoxanthomonas* (up to 13.1%), *Zavarzinella* (up to 11.8%), *Vulgatibacter* (up to 11.6%), *Sorangium* (up to 10.6%), *Janthinobacterium* (up to 8.4%), *Promicronospora* (up to 7.4%), and *Ramlibacter* (up to 7.4%) were the most abundant within the top25 genera, accounting for 54–64% of the total microbial community of the metaproteome (Supporting Information, Figure S2). The changes in relative abundance with respect to optimal irrigation with high quality water (TW-C) were calculated at the level of the phyla and the top25 genera (Fig. 4). Overall, the relative abundance of proteins from dominant groups was different in TW-RDI and RW-RDI. The relative abundance of proteins affiliated to *Actinobacteria*, *Gemmatimonadetes*, *Planctomycetes*, and *Proteobacteria* was higher in TW-C than in RW-C, while proteins affiliated to *Acidobacteria* were more adundant when the soil was irrigated with RW-C. Regarding water quantity, proteins affiliated to *Planctomycetes* and *Proteobacteria* were found when TW-RDI was applied, whereas proteins from *Acidobacteria*, *Actinobacteria*, and *Bacteroidetes* were found in August for the RW-RDI treatment. At the level of microbial genera, proteins affiliated to *Belnapia*, *Brevundimonas*, *Gemmatirosa* (except in June), *Geobacter*, *Rubrobacter*, *Solirubrobacter*, and *Zavarzinella* (except in January) were dominant when soil was irrigated with TW-C. In contrast, proteins affiliated to *Altibacter*, *Enterococcus*, *Ilumatobacter*, *Pedobacter*, *Pyronomonas*, *Ramlibacter*, and *Thermoanaerobaculum* were found with RW-C. The TW-RDI treatment led to an increase in proteins from *Ilumatobacter*, *Rubrobacter*, and *Vulgatibacter*, while proteins from *Promicronospora*, *Sorangium*, and *Zavarzinella* (except in January) were found in TW-C. In relation to the quantity of reclaimed water supplied, proteins from *Altibacter*, *Haliangium*, *Promicronospira*, *Pyrinomonas*, *Ramlibacter*, *Thermoanaerobaculum*, and *Vulgatibacter* were found for RW-C, while RW-RDI gave rise to proteins affiliated with *Gemmatirosa*, *Geobacter*, *Pseudoxanthomonas*, *Segetibacter*, and *Zavarzinell*a.

### Functional diversity

The higher abundance of proteins was analyzed at the genus level, analyzing water transferred from the river (Table 3) and reclaimed water (Table 4). For this, the protein had to be both abundant in at least two out of three replicates and solely abundant in one treatment. Generally, the proteins showed three differential patterns with regard to RDI: immediate, delayed, or ubiquitous expression. For TW-C, immediately abundant proteins included peroxiredoxin (COG1225) in *Azospirillum*, *Caenimonas*, *Sorangium*, and *Vulgatibacter* and Mn-containing catalase (COG3546) in *Ramlibacter* and *Sorangium*. Proteins such as rotamase (COG0652) in *Pseudoxanthomonas* were abundant during the later stages for TW-C, whereas (for example) 3-hydroxyacyl-CoA dehydrogenase (COG1250) in *Altibacter*, *Pseudoxanthomonas*, *Solirubrobacter*, and *Vulgatibacter* and ribulose 1,5-bisphosphate carboxylase (RuBisCO, COG1850) in *Rubrobacter* were abundant in all seasons (Table 3). The abundance pattern was similar for irrigation with TW-RDI, RW-C, and RW-RDI (Table 4). On RW-RDI, catalase (COG0753) in *Ramlibacter* and *Promicronospora* was abundant in August 2014 and June 2015. Further, glutaminase (COG2066) in *Vulgatibacter* and aconitase A (COG1048) in *Brevundimonas* and *Vulgatibacter* were abundant in August 2014 and January 2015. 3-hydroxyacyl-CoA dehydrogenase (COG1250) in *Pedobacter*, glutamine synthetase (COG0174) in *Labilithrix*, ribulose 1,5-bisphosphate carboxylase (COG1850) in *Rubrobacter* and *Zavarzinella* and superoxide dismutase (COG0605) in *Pyrinomonas* and *Rubrobacter* were only abundant in August 2014 on RW-RDI. On RW-C, Mn-containing catalase (COG3546) in *Ilumatobacter* and *Niabella* was abundant in all seasons while yuperoxide dismutase (COG0605) in *Pyrinomonas* and *Rubrobacter* was found in January and June 2015. Aconitase A (COG1048) in *Sorangium*, *Streptomyces* and *Terrimonas* was abundant in August 2014 and January 2015 while ribulose 1,5-bisphosphate carboxylase (COG1850) in *Novosphingobium* was only abundant in January 2015 (Table 4).

**Table 3:** Preferred abundance of proteins during irrigation with transfer water. Optimal amounts (TW-C) and regulated deficit irrigation during summer (TW-RDI) are indicated.

**Function**	**Accession**	**Aug'14**	**Jan'15**	**Jun'15**	**Organism(s)**
ABC-type sugar transport system, periplasmic component	COG1653	TW-RDI	TW-RDI	TW-RDI	*Acidithrix, Azospirillum, Moorella, Pyrinomonas, Sediminibacterium*
Acetyl-CoA acetyltransferase	COG0183	TW-RDI	TW-RDI	TW-RDI	*Segetibacter*
Aconitase A	COG1048	TW-RDI	TW-RDI	TW-RDI	*Gemmatirosa, Pseudoxanthomonas, Solirubrobacter, Terrimonas, Zavarzinella*
Adenylylsulfate kinase and related kinases	COG0529	TW-RDI	TW-RDI	TW-RDI	*Rubrobacter, Sorangium*
Bacterial nucleoid DNA-binding protein	COG0776	TW-RDI	TW-RDI	TW-RDI	*Niastella, Rubrobacter, Sorangium*
Dihydroxyacid dehydratase/phosphogluconate dehydratase	COG0129	TW-RDI	TW-RDI	TW-RDI	*Zavarzinella*
Glutamine synthetase	COG0174	TW-RDI	TW-RDI	TW-RDI	*Janthinobacterium, Promicromonospora, Pseudoxanthomonas, Segetibacter, Solirubrobacter, Vulgatibacter*
Microcystin-dependent protein	COG4675	TW-RDI	TW-RDI	TW-RDI	*Acidithrix, Pedobacter*
Mn-containing catalase	COG3546	TW-RDI	TW-RDI	TW-RDI	*Ilumatobacter, Pedobacter, Promicromonospora, Rubrobacter, Solirubrobacter, Zavarzinella*
Opacity protein and related surface antigens	COG3637	TW-RDI	TW-RDI	TW-RDI	*Janthinobacterium, Pseudoxanthomonas, Thermoanaerobaculum*
S-adenosylhomocysteine hydrolase	COG0499	TW-RDI	TW-RDI	TW-RDI	*Aeromicrobium, Janthinobacterium, Pedobacter, Sorangium, Thermoanaerobaculum, Vulgatibacter*
Malate/lactate dehydrogenases	COG0039		TW-RDI	TW-RDI	*Caenimonas, Janthinobacterium, Sorangium*
Peroxiredoxin	COG1225		TW-RDI	TW-RDI	*Rubrobacter, Sorangium, Vulgatibacter, Zavarzinella*
TRAP-type mannitol/chloroaromatic compound transport system	COG4663			TW-RDI	*Acidithrix*
ABC-type amino acid transport/signal transduction systems	COG0834	TW-RDI	TW-RDI		*Jiangella, Pseudoxanthomonas*
ABC-type branched-chain amino acid transport systems, periplasmic component	COG0683	TW-RDI	TW-RDI		*Niastella*
ABC-type nitrate/sulfonate/bicarbonate transport systems, periplasmic components	COG0715	TW-RDI	TW-RDI		*Deferrisoma, Pseudoxanthomonas*
ABC-type oligopeptide transport system, periplasmic component	COG4166	TW-RDI	TW-RDI		*Rubrobacter*
Anaerobic dehydrogenases, typically selenocysteine-containing	COG0243	TW-RDI	TW-RDI		*Arenimonas, Pyrinomonas*
Catalase	COG0753	TW-RDI	TW-RDI		*Promicromonospora, Thermobaculum*
Dienelactone hydrolase and related enzymes	COG0412	TW-RDI	TW-RDI		*Vulgatibacter*
Enoyl-[acyl-carrier-protein] reductase (NADH)	COG0623	TW-RDI	TW-RDI		*Caenimonas*
Glyceraldehyde-3-phosphate dehydrogenase/erythrose-4-phosphate dehydrogenase	COG0057	TW-RDI	TW-RDI		*Pedobacter, Pseudoxanthomonas, Rubrobacter, Sorangium, Vulgatibacter*
Monomeric isocitrate dehydrogenase	COG2838	TW-RDI	TW-RDI		*Altibacter, Jiangella, Segetibacter*
Nucleoside diphosphate kinase	COG0105	TW-RDI	TW-RDI		*Brevundimonas*
RecA/RadA recombinase	COG0468	TW-RDI	TW-RDI		*Pseudoxanthomonas, Vulgatibacter*
TRAP-type C4-dicarboxylate transport system, periplasmic component	COG1638	TW-RDI	TW-RDI		*Aureimonas, Niastella, Pedobacter*
5'-nucleotidase/2',3'-cyclic phosphodiesterase and related esterases	COG0737		TW-RDI		*Solirubrobacter*
Aerobic-type carbon monoxide dehydrogenase, large subunit CoxL/CutL homologs	COG1529		TW-RDI		*Pyrinomonas*
Aspartate-semialdehyde dehydrogenase	COG0136		TW-RDI		*Ramlibacter*
Carbon dioxide concentrating mechanism/carboxysome shell protein	COG4577		TW-RDI		*Ramlibacter*
Glutaminase	COG2066		TW-RDI		*Sorangium*
Threonine dehydrogenase and related Zn-dependent dehydrogenases	COG1063		TW-RDI		*Niastella*
Glucose/sorbosone dehydrogenases	COG2133	TW-RDI			*Gemmatirosa*
MoxR-like ATPases	COG0714	TW-RDI			*Acidithrix*
3-hydroxyacyl-CoA dehydrogenase	COG1250	TW-C	TW-C	TW-C	*Altibacter, Pseudoxanthomonas, Solirubrobacter, Vulgatibacter*
ABC-type amino acid transport/signal transduction systems	COG0834	TW-C	TW-C	TW-C	*Pseudoxanthomonas, Terrimonas*
ABC-type sugar transport system, periplasmic component	COG1653	TW-C	TW-C	TW-C	*Rhodopirellula, Rubrobacter, Pedobacter, Pyrinomonas, Sediminibacterium*
Aconitase A	COG1048	TW-C	TW-C	TW-C	*Gemmatimonas, Pedobacter, Solirubrobacter, Sorangium, Zavarzinella*
Glutamine synthetase	COG0174	TW-C	TW-C	TW-C	*Janthinobacterium, Pedobacter, Promicromonospora, Thermoanaerobaculum, Solirubrobacter*
Malate/lactate dehydrogenases	COG0039	TW-C	TW-C	TW-C	*Janthinobacterium, Pseudoxanthomonas, Sorangium, Sphaerobacter, Vulgatibacter*
RecA/RadA recombinase	COG0468	TW-C	TW-C	TW-C	*Arenimonas, Pseudoxanthomonas, Vulgatibacter, Zavarzinella*
Ribulose 1,5-bisphosphate carboxylase, large subunit	COG1850	TW-C	TW-C	TW-C	*Rubrobacter*
5'-nucleotidase/2',3'-cyclic phosphodiesterase and related esterases	COG0737		TW-C	TW-C	*Pseudoxanthomonas, Solirubrobacter, Zavarzinella*
Bacterial nucleoid DNA-binding protein	COG0776		TW-C	TW-C	*Brevundimonas, Rubrobacter, Sorangium, Vulgatibacter*
Isocitrate dehydrogenases	COG0538		TW-C	TW-C	*Pyrinomonas, Vulgatibacter, Zavarzinella*
Superoxide dismutase	COG0605		TW-C	TW-C	*Pyrinomonas*
Microcystin-dependent protein	COG4675	TW-C		TW-C	*Acidithrix*
Nucleoside diphosphate kinase	COG0105			TW-C	*Solirubrobacter*
Peptidyl-prolyl cis-trans isomerase (rotamase) - cyclophilin family	COG0652			TW-C	*Pseudoxanthomonas*
ABC-type branched-chain amino acid transport systems, periplasmic component	COG0683	TW-C	TW-C		*Flavobacterium, Sorangium*
ABC-type nitrate/sulfonate/bicarbonate transport systems, periplasmic components	COG0715	TW-C	TW-C		*Janthinobacterium, Sorangium, Sphaerobacter, Vulgatibacter*
Arylsulfatase A and related enzymes	COG3119	TW-C	TW-C		*Niastella, Rubrobacter*
Glutaminase	COG2066	TW-C	TW-C		*Janthinobacterium*
Glyceraldehyde-3-phosphate dehydrogenase/erythrose-4-phosphate dehydrogenase	COG0057	TW-C	TW-C		*Pseudoxanthomonas, Pyrinomonas, Sediminibacterium, Vulgatibacter*
Catalase	COG0753		TW-C		*Niastella, Ramlibacter, Solimonas*
Glycerol uptake facilitator and related permeases (Major Intrinsic Protein Family)	COG0580		TW-C		*Promicromonospora, Sphaerobacter*
L-alanine-DL-glutamate epimerase and related enzymes of enolase superfamily	COG4948		TW-C		*Sphingomonas*
Peroxiredoxin	COG1225		TW-C		*Azospirillum, Caenimonas, Sorangium, Vulgatibacter*
ABC-type dipeptide transport system, periplasmic component	COG0747	TW-C			*Haliangium*
Aspartate-semialdehyde dehydrogenase	COG0136	TW-C			*Ramlibacter*
Carbon dioxide concentrating mechanism/carboxysome shell protein	COG4577	TW-C			*Ramlibacter*
Cbb3-type cytochrome oxidase, cytochrome c subunit	COG2993	TW-C			*Pseudoxanthomonas*
Mn-containing catalase	COG3546	TW-C			*Niabella, Zavarzinella*
MoxR-like ATPases	COG0714	TW-C			*Ramlibacter, Sorangium*
TRAP-type C4-dicarboxylate transport system, periplasmic component	COG1638	TW-C			*Ramlibacter, Rubrobacter*

**Table 4:** Preferred abundance of proteins during irrigation with reclaimed water. Optimal amounts (RW-C) and regulated deficit irrigation during summer (RW-RDI) are indicated.

**Function**	**Accession**	**Aug'14**	**Jan'15**	**Jun'15**	**Organism(s)**
Catalase	COG0753	RW-RDI		RW-RDI	*Promicromonospora, Ramlibacter*
Opacity protein and related surface antigens	COG3637	RW-RDI		RW-RDI	*Azospirillum, Pseudoxanthomonas*
Microcystin-dependent protein	COG4675	RW-RDI		RW-RDI	*Pedobacter, Ramlibacter*
5'-nucleotidase/2',3'-cyclic phosphodiesterase and related esterases	COG0737	RW-RDI		RW-RDI	*Pseudoxanthomonas*
Mn-containing catalase	COG3546			RW-RDI	*Ramlibacter*
Glutaminase	COG2066	RW-RDI	RW-RDI		*Vulgatibacter*
ABC-type oligopeptide transport system, periplasmic component	COG4166	RW-RDI	RW-RDI		*Pedobacter*
Aconitase A	COG1048	RW-RDI	RW-RDI		*Brevundimonas, Vulgatibacter*
L-alanine-DL-glutamate epimerase and related enzymes of enolase superfamily	COG4948	RW-RDI			*Altibacter*
S-adenosylhomocysteine hydrolase	COG0499	RW-RDI			*Janthinobacterium*
Bacterial nucleoid DNA-binding protein	COG0776	RW-RDI			*Altibacter*
3-hydroxyacyl-CoA dehydrogenase	COG1250	RW-RDI			*Pedobacter*
Glutamine synthetase	COG0174	RW-RDI			*Labilithrix*
TRAP-type mannitol/chloroaromatic compound transport system, periplasmic component	COG4663	RW-RDI			*Aureimonas, Jiangella*
Glyceraldehyde-3-phosphate dehydrogenase/erythrose-4-phosphate dehydrogenase	COG0057	RW-RDI			*Pseudoxanthomonas*
MoxR-like ATPases	COG0714	RW-RDI			*Sediminibacterium*
Acetoacetate decarboxylase	COG4689	RW-RDI			*Caenimonas*
TRAP-type C4-dicarboxylate transport system, periplasmic component	COG1638	RW-RDI			*Aureimonas*
Ribulose 1,5-bisphosphate carboxylase, large subunit	COG1850	RW-RDI			*Rubrobacter, Zavarzinella*
Arylsulfatase A and related enzymes	COG3119	RW-RDI			*Pedobacter, Rubrobacter*
Isocitrate dehydrogenases	COG0538	RW-RDI			*Zavarzinella*
Superoxide dismutase	COG0605	RW-RDI			*Pyrinomonas, Rubrobacter*
Peroxiredoxin	COG1225	RW-RDI			*Vulgatibacter*
ABC-type sugar transport system, periplasmic component	COG1879	RW-RDI			*Acidithrix, Moorella, Sphingomonas*
Malate/lactate dehydrogenases	COG0039	RW-RDI			*Pyrinomonas, Sorangium*
ABC-type nitrate/sulfonate/bicarbonate transport systems, periplasmic components	COG0715	RW-RDI			*Pseudoxanthomonas, Sorangium, Zavarzinella*
Bacterial nucleoid DNA-binding protein	COG0776	RW-C	RW-C	RW-C	*Flavobacterium, Pseudoxanthomonas*
Dihydroxyacid dehydratase/phosphogluconate dehydratase	COG0129	RW-C	RW-C	RW-C	*Acidithrix*
Mn-containing catalase	COG3546	RW-C	RW-C	RW-C	*Ilumatobacter, Niabella*
ABC-type dipeptide transport system, periplasmic component	COG0747		RW-C	RW-C	*Sorangium*
Microcystin-dependent protein	COG4675		RW-C	RW-C	*Ramlibacter*
MoxR-like ATPases	COG0714		RW-C	RW-C	*Niastella, Sorangium*
S-adenosylhomocysteine hydrolase	COG0499		RW-C	RW-C	*Zavarzinella*
Superoxide dismutase	COG0605		RW-C	RW-C	*Pyrinomonas, Rubrobacter*
Acetyl-CoA acetyltransferase	COG0183	RW-C		RW-C	*Promicromonospora*
Opacity protein and related surface antigens	COG3637	RW-C		RW-C	*Jiangella, Pseudoxanthomonas*
Histone H4	KOG3467			RW-C	*Aureimonas*
TRAP-type C4-dicarboxylate transport system, periplasmic component	COG1638			RW-C	*Vulgatibacter*
Glyceraldehyde-3-phosphate dehydrogenase/erythrose-4-phosphate dehydrogenase	COG0057	RW-C	RW-C		*Sorangium, Vulgatibacter*
Arylsulfatase A and related enzymes	COG3119	RW-C	RW-C		*Niastella*
Aconitase A	COG1048	RW-C	RW-C		*Sorangium, Streptomyces, Terrimonas*
ABC-type nitrate/sulfonate/bicarbonate transport systems, periplasmic components	COG0715		RW-C		*Janthinobacterium*
ABC-type sugar transport system, periplasmic component	COG1653		RW-C		*Azospirillum*
Anaerobic dehydrogenases, typically selenocysteine-containing	COG0243		RW-C		*Pyrinomonas*
Enoyl-[acyl-carrier-protein] reductase (NADH)	COG0623		RW-C		*Caenimonas*
Glutaminase	COG2066		RW-C		*Sorangium*
Glutamine synthetase	COG0174		RW-C		*Promicromonospora*
Ribosome recycling factor	COG0233		RW-C		*Segetibacter*
Ribulose 1,5-bisphosphate carboxylase, large subunit	COG1850		RW-C		*Novosphingobium*
3-hydroxyacyl-CoA dehydrogenase	COG1250	RW-C			*Vulgatibacter*

## Discussion

### The impacts of reclaimed water and reduced irrigation on the activity of soil microbial community

The phylogenetic analysis of proteins and the quantification of their abundance can constitute a valuable approach for the analysis of active microbial populations and it has been demonstrated that these populations are strongly connected to ecosystem processes such as nutrient cycling^25, 31^. Several studies have revealed the impacts of drought on the composition of the soil microbial community and soil organic matter cycling^18, 32–34^. However, the impacts of reduced irrigation on the activity of soil microbial populations have not been evaluated so far. Several studies observed that the diversity of the soil microbial community increases under drought and this phenomenon was due to the fact that drought moderates competition within the microbial community^35, 36^. Here, contrarily to hypothesized, ANOVA revealed that reduced irrigation (water quantity) did not influence the active phylogenetic diversity of soil. However, the phylogenetic and functional diversity were negatively affected by irrigation with RW in January and June (both 2015), but not in August 2014. The elevated salinity (high EC value) and DOC content of reclaimed water could have promoted the selection of less diverse, yet more specialized, microbial communities, as observed by genomic approaches^37, 38^. Interestingly, the lower active phylogenetic and functional diversities in soils irrigated with RW agree with the low respiration values. These results suggest that the utilization of reclaimed water affected the activity of the soil microbial community and its C cycling^39, 40^. Most of the identified proteins are involved in cellular metabolism and the reduced functional diversity index in RW-irrigated soils may indicate that reclaimed water alters cellular metabolism which translate into decreased soil respiration. Interestingly, this is occurring in RW-treated soils despite of reclaimed water containing soluble OM that could potentially act as substrate for mineralization. These results reinforce the negative impact of high EC in the overall activity of the soil microbial community^39^. Furthermore, it can be suggested that the activity of soil microbial populations was strongly shaped by the high EC and salinity content of reclaimed water. For instance, the activity of *Haliangium* sp., a halophite which grows on up to 5% NaCl^41^, was greater in RW-C than in TW-C, particularly in August 2014. Overall, the significant impact of reclaimed water in the composition of the active soil microbial community was visualized by the PCA in August-samples, where RW-C and RW-RDI samples were separated from samples that received water with optimal quality (TW-C and TW-RDI). Several studies have observed an impact of salinization in the composition of the soil microbial community^37, 38^.

Reduced irrigation caused changes in the composition and functionality of the active microbial community. For instance, PCA revealed that the composition and functionality of the active microbial community were different in TW-RDI soil in August 2014 in comparison to that in TW-C soil samples. Nevertheless, ANOVA indicated that water quantity (i.e. reduced irrigation) did not impact the active phylogenetic diversity. Changes in community composition could maintain the level of phylogenetic diversity at the same level than soils receiving optimal amount of water. Indeed, an alteration of the total microbial community composition in drought impacted soils was previously reported^18^. Accordingly, changes in community composition could explain the low soil respiration of TW-RDI in August 2014 despite DOC content was not significantly different between TW-C and TW-RDI. Overall, these results support the idea that changes in community composition rather than in diversity are of the greatest importance for soil respiration^42, 43^. The TW-RDI treatment induced activity of *Acidobacteria* and *Bacteroidetes*, while RW-RDI promoted the activity of proteobacterial phylotypes. By contrast, Barnard *et al*.^32^ observed that *Acidobacteria* were negatively influenced by water limitation^32^. The higher content of soluble OM in RW may benefit the activity of copiotrophs, such as some *Proteobacteria*, when water reductions are too severe, improving the resistance of soils irrigated with RW. Besides changes in community composition, the lower respiration in RDI samples can be due to the fact that drought reduces pore connectivity and the diffusion of substrates, compromising the activity of the soil microbial community^44, 45^.

Altogether, metaproteomics enabled the molecular study of the functional mechanisms implied in the adaptations of bacteria to reduced irrigation. Many bacteria, including *Proteobacteria*, preferentially utilize tricarboxylic acid (TCA) cycle intermediates - such as the C_4_-dicarboxylates malate, fumarate, and succinate - as carbon and energy sources. Certain transport mechanisms are involved in C_4_-utilization, such as TRAP-type C_4_-dicarboxylate transport or the ABC-type sugar transport systems^46^. Here, we found that these proteins were expressed solely during the RDI event (August) in some proteobacterial populations - such as *Sphingomonas* sp. and *Aureimonas* sp. – that were growing in soils irrigated with RW. This result could indicate that the copiotrophism mediated by transport of C compounds enhances the resistance against reduced irrigation.

Other mechanisms which aid resistance against water deficit may involve C-fixation through the expression of ribulose 1,5-bisphosphate carboxylase (RubisCO), in *Rubrobacter* (*Actinobacteria*) and *Zavarzinella* (*Planctomycetes*). Indeed, RubiscCO genes have been found to be widespread, phylogenetically, including actinobacterial populations^47^. Furthermore, water stress may cause an increase in the content of reactive oxygen species (ROS) in plant cells, which is confronted by the increased production or activity of enzymatic systems such as superoxide dismutase (SOD)^48^. We found that SOD was more abundant in *Pyrimonas* and *Rubrobacter* during RDI. This result suggests that: i) plants and bacteria might share evolutionarily-conserved molecular mechanisms for fighting the production of ROS under stress conditions (i.e. drought); and ii) these mechanisms could explain the success of some actinobacterial groups, such as *Rubrobacter* sp., under water restrictions - in fact, it has been observed that *Actinobacteria* are able to maintain their activity and growth under drought conditions^18^.

### The resilience of soil microbial community after reduced irrigation

As described above, RDI in summer lowered soil respiration, in comparison to soils that received an optimal amount of water (TW-C and RW-C). But, what are the consequences of reduced irrigation in the following seasons? Soil respiration of RDI-treated soils increased once the optimal water amount was resupplied (January and June 2015), particularly when reclaimed water was utilized (RW-RDI). This recovery of soil respiration (functional resilience) after RDI was not associated with an increase in the phylogenetic diversity of the active community, but it was linked to a reduction of functional diversity (particularly in June). It is known that a multitude of microbial populations carry out the same generalist processes such as soil respiration^26, 49^. This functional redundancy can explain the fact that soil respiration recovered after RDI while the active phylogenetic diversity did not. Moreover, changes in the composition of the active microbial community occurred once the optimal water amount was resupplied, as revealed by the PERMANOVA. For instance, the activity of some populations increased in January and June in comparison to that in August. This was the case of members of the *Firmicutes* such as *Enterococcus*, for *Proteobacteria* such as *Nitrosomonadales*, *Sphingomonadales*, and *Xanthomondales*, and *Actinobacteria* such as *Rubrobacterales*, *Solirubrobacterales*, and *Planctomycetales*. It has been suggested that the resilience of the soil microbial community to drought increases with increasing the abundance of bacteria that can be classified as copiotrophs, such as many *Proteobacteria* ^50, 51^. Indeed, DOC was the greatest in RW-RDI in June. This C fraction contains soluble OM and available energy that can be rapidly utilized by copiotrophs^31^. Moreover, the capacity of endosporulation under harsh conditions of some Gram-positive bacteria such as *Firmicutes* may be related to their capacity for recovery after RDI^8^.

Metaproteomics allowed the deciphering of molecular mechanisms that favor the resilience of bacterial populations after reduced irrigation. Some bacterial populations expressed proteins exclusively in January and June and these proteins are potentially involved in the resilience of such groups. For instance, peroxiredoxin from *Rubrobacter* sp, *Sorangium* sp, *Vulgatibacter*, and *Zavarzinella* was found in January and June TW-RDI samples. Peroxiredoxin plays major roles in the prevention of oxidative damage and it has been shown to be overexpressed after water stress and to be involved in drought tolerance in plants^52^. In the same way, threonine dehydrogenase was found to increase after RDI in TW-irrigated soils. This enzyme has been described as a putative drought tolerance gene in plants^53^. These proteins illustrate the inter-kingdom molecular conserved mechanisms for fighting against water stresses. Additionally, mechanisms that involve C-fixation through the carboxysomes (bacterial organelles consisting of polyhedral protein shells filled with the enzyme RubisCO) can be considered a resilience mechanisms of *Ramlibacter* sp. (*Proteobacteria*) that could increase its activity at the expense of C-fixation in stages when there is most likely to be high competition for growth (restored water supply). Moreover, the expression of microbial catalase genes, among others, has been found to be involved in the response to reactive-oxygen species (ROS)^54^. Aerobic conditions, as found in dry soils^34^, could promote the production of ROS and the consequent expression of bacterial catalases. Here, we found the Mn-containing catalase in *Ilumatobacter*, *Pedobacter*, *Promicromonospora*, *Rubrobacter*, *Solirubrobacter*, and *Zavarzinella* at all seasons in RW-RDI and in *Niabella*, *Zavarzinella* in August 2014 in RW-C.

Metaproteomics allowed a better understanding of the responses of soil microbial communities to water managements that are designed to withstand the deficiency of water resources, as well as the molecular basis of these adaptations. Bacterial populations displayed molecular mechanisms for combating water stresses that are conserved in plants in evolutionary terms. These molecular mechanisms are related to the transformation of ROS and the enhancement of organic C input to the cell. According to the proposed hypothesis, we conclude that:

i) Reduced irrigation decreased the functional diversity of the soil microbial community but did not reduce its active phylogenetic diversity. Changes in the composition of the active community explained the stability of phylogenetic diversity in RDI soil samples.
ii) Active phylogenetic diversity was not affected by RDI in August. However, functional diversity was lowered in RW-RDI in August and it was not resilient once the water quantity was resupplied.
iii) Soil respiration experienced a recovery once water amount was resupplied, particularly when reclaimed water was utilized (RW-RDI). This recovery was related to changes in the composition of the active soil microbial community with increased activity of some *Proteobacteria* and *Actinobacteria* populations.

This study illustrates how soil metaproteomics can help to understand the responses of soil microbial communities to water management, as well as their ecological attributes such as resistance and resilience. In episodes of reduced water availability, the RW-RDI irrigation system would be necessary. This treatment induced changes in the diversity of the soil microbial community probably due to the high electrical conductivity of RW (high salt content). However, RW-RDI promoted a resilient soil respiration (probably due to the high DOC content) that can contribute to the maintenance of soil fertility and crop productivity.

## Material and Methods

### Experimental design, irrigation treatments, and soil sampling

The experiment was developed in a commercial 0.5-ha orchard cultivated with 11-year-old ‘Star Ruby’ grapefruit trees (*Citrus paradisi* Macf) grafted on Macrophylla rootstock (*Citrus macrophylla*). This orchard is located in Campotéjar-Murcia, Spain (38°07′18”N; 1°13′15”W) and is characterized by a Mediterranean semiarid climate with warm, dry summers and mild winter conditions. In 2014 and 2015, the average annual reference evapotranspiration (ET_0_) and rainfall are 1326 and 300 mm, respectively. The soil within the first 90 cm depth had a loamy texture (24% clay, 33% loam, and 43% sand), with an average bulk density of 1.41 g cm^−3^. The content of total organic C and total N in soil was 15.30 and 1.98 g kg^−1^, respectively.

The experiment took the form of three completely randomized plots per irrigation treatment (for a total of 12 plots). Each plot was made up of 12 trees (288 m^2^). Four irrigation treatments, based on the water quality of the irrigation source and the water quantity, were performed. One source (TW), with an average electrical conductivity (EC) of 1.0 dS m^−1^, was pumped from the “Tagus-Segura” water transfer canal, which supplies the major part of the water used for irrigation in south-east Spain. The other source was tertiary reclaimed water (RW), pumped from a nearby wastewater treatment plant. This source was automatically blended, at the irrigation control-head, with water from the canal to maintain a constant EC of around 3 dS m^−1^ throughout the experiment.

From 2005 to 2007 the whole orchard was fully irrigated with water transferred from the channel (TW). The following irrigation scheme was performed from 2008 onwards. The control treatments involved irrigation with TW or RW during the whole season at 100% of the crop evapotranspiration (ETc) (TW-C and RW-C, respectively). The RDI treatment consisted of irrigation at 100% ETc, except during the second stage of fruit growth, for 55–65 days between late June and mid-September, when it consisted of 50% of the amount of water applied to the control (TW-RDI and RW-RDI). This means that the TW-RDI and RW-RDI soils sampled in August 2014 had received 50% of the water utilized in TW-C and RW-C during approximately two months of each of the previous seven years. In both years, the average amounts of water applied were measured with inline water flow meters and in the control treatments reached 614 (TW-C) and 593 (RW-C) mm, and in the RDI treatments 518 (TW-RDI) and 493 (RW-RDI) mm, respectively, Hence, the RDI treatments meant reductions of about 16% in the volume of water applied per year. All treatments included application of the same amounts of fertilizer (N–P_2_O_5_–K_2_O), applied through the drip irrigation system: 215–110–150 kg ha^−1^ year^−1^. Nitrogen was applied in the forms of 50% nitric and 50% ammonium. Pest control practices and pruning were those commonly used by growers in the area, and no weeds (glyphosate, 36%) were allowed to develop within the orchards. The following insecticides were utilized: piriproxifen (10%), abamectin (1.8%) and hexitiazox (10%). The following fungicides were applied: mancozeb (80%) and oxychloride copper (50%). In 2015, the yield for all irrigation treatments was 233, 184, 244 and 209 kg fruit tree^−1^ for TW-C, TW-RDI, RW-C and RW-RDI, respectively^8^.

A soil sample was taken from each of the 12 plots in August 2014, January 2015, and June 2015, from the first 20 cm depth. Each sample was taken under the canopy of one tree and was composed of six subsamples. The samples were sieved at <2 mm. The contents of sand, silt, and clay of the soil were 50, 26, and 24%, respectively, with an average bulk density of 1.37 g cm^−3^. The soil was classified as a Typic Haplocalcid, according to Soil Survey Staff^55^. A fraction of each sample was kept at room temperature for chemical analysis, another fraction was stored at 4 °C until the chemical analyses and respiration assays were performed, and the rest was stored at −80 °C for proteomic analyses.

### Soil chemical analyses and soil respiration

The total nitrogen (N) and total organic C contents were determined using an Elemental Analyzer (C/N Flash EA 112 Series-Leco Truspec). Electrical conductivity and pH were measured in a 1:5 (w:v) aqueous solution in a Conductivimeter and Crison mod 2001pH meter (Crison Instruments, Barcelona, Spain), respectively. The dissolved organic C (DOC) of the soil was extracted with distilled water (1:5, w:v) by shaking for 2 h, followed by centrifugation at 13,000 rpm for 15 min and filtration. The analysis of the C content in the extracts was performed in an analyzer for liquid samples (Multi N/C 3100, Analytik Jena).

Microbial respiration (CO_2_ emission) was measured in 10-ml capped tubes containing one gram of soil. The soil samples were moistened with distilled water to 60% of their water-holding capacity. The vials were then sealed hermetically and incubated in the dark at 28 °C for 19 days. The concentration of CO_2_ was analyzed periodically with a gas chromatograph (Trace Ultra Thermo Scientific, Milan, Italy) with a packed column (Trace PLOT TG-BOND Q GC, Trace Ultra Thermo Scientific)^56^.

### Water characterization

An inductively coupled plasma mass spectrometer (ICP-ICAP 6500 DUO Thermo, UK) was used to determine the concentrations of Na, K, B, Ca and Mg. Anions (Cl, NO_3_^−^, PO_4_^3−^ and SO_4_^2−^) were analyzed by ion chromatography, with a liquid chromatograph (Metrohm, Switzerland). The analysis of the C and N content in water samples was performed in an analyzer for liquid samples (Multi N/C 3100, Analytik Jena). Electrical conductivity (EC_w_) and pH were measured in a Crison mod. Conductivimeter and a Crison mod 2001pH meter (Crison Instruments, Barcelona, Spain), respectively.

### Preparation of the semiarid soil metagenome

The DNA from the soil samples obtained in this study, together with that of other semiarid soils^13^, was utilized for the preparation of a semiarid soil metagenome. A TruSeq PCR Free LT Sample Preparation Kit (Illumina, San Diego, CA, USA) was used for library preparation. The library size-distribution was checked on an Agilent 2100 Bionalyser (Agilent Technologies). The libraries were sequenced on an Illumina HiSeq. 2000 at Brigham Young University, Provo, UT, USA. The reads were quality trimmed by removing adapters with Trimmomatic (v 0.27), using ILLUMINA TRUSEQ. 2-PE adapters with a seed mismatch threshold, palindrome clip threshold, and simple clip threshold set at 2, 30, and 10 respectively^57^. Furthermore, the sequencing reads were filtered and the resulting sequences were normalized using methods previously described^58, 59^ and Khmer (v 0.7.1), and command normalise-by-median.py. Next, errors were trimmed by removing low abundance fragments of high coverage reads with Khmer and command filter-abund.py −V. The paired-end assembly of the remaining reads was performed with MEGAHIT (v1.0.2, with default parameters)^60^. The sequence data of all contig sequences have been deposited in the MG RAST data set (number 4697967.3) (http://metagenomics.anl.gov/linkin.cgi?metagenome=4697967.3).

### Protein extraction and sample processing

Protein extraction of all soil samples (n = 36) was performed as described by Chourey and colleagues^21^, a method which was found to be suitable for semiarid soils^20^. Cell lysis and disruption of soil aggregates were performed by boiling at 100 °C for 10 min in SDS-buffer. The concentration and purification of proteins were performed using TCA at 25% and three washing steps with 1.0 ml of chilled 100% acetone each. The protein pellets were re-suspended in 20 μl of SDS lysis buffer (100 mM Tris/HCl, 4% SDS, and 0.1 mM DTT) and incubated at 95 °C for 5 min. The whole proteins protein lysates were loaded on SDS gels (4% stacking gel, 12% separating gel) and electrophoresis was performed until 0.5 cm into the gel, at 20 mA. Afterwards, the gels were stained with colloidal Coomassie brilliant blue and gel-pieces were treated and digested as described elsewhere^22^.

### Mass spectrometric analysis

The normalization of the peptide content of samples was done using desalting devices (ZipTip) with a maximum loading capacity of 5 µg per tip. The peptide lysates were desalted and reconstituted in 0.1% formic acid. Peptide lysates were separated on a UHPLC system (Ultimate 3000 RSLCnano, Dionex/Thermo Fisher Scientific, Idstein, Germany). The samples (5 µL) were first loaded for 5 min on the pre-column with a maximum loading capacity of 2 µg (µ-precolumn, Acclaim PepMap, 75 µm inner diameter, 2 cm, C18, Thermo Scientific), at 4% mobile phase B (80% acetonitrile in nanopure water with 0.08% formic acid) and 96% mobile phase A (nanopure water with 0.1% formic acid), and then were eluted from the analytical column (PepMap Acclaim C18 LC Column, 25 cm, 3 µm particle size, Thermo Scientific) over a 120-min linear gradient of mobile phase B (4–55% B). Mass spectrometry was performed on a Q Exactive HF mass spectrometer (Thermo Fisher Scientific, Waltham, MA, USA) with a TriVersa NanoMate (Advion, Ltd., Harlow, UK) source in LC-chip coupling mode.

### LC-MS data analysis

Proteome Discoverer (v1.4, Thermo Scientific) was used for protein identification and the MS/MS spectra acquired were searched with Sequest HT against the specific metagenome obtained for semiarid soils. Enzyme specificity was selected as trypsin with up to two missed cleavages allowed, using 10 ppm peptide ion tolerance and 0.05 Da MS/MS tolerances. Oxidation at methionines and carbamylation at lysines and arginines were selected as the variable modifications and carbamidomethylation at cysteines as the static modification. Only peptides with a false discovery rate (FDR) below 0.01, as calculated by Percolator^61^, and a peptide rank of one were considered as identified. The rank one peptide is the highest scoring match to a particular query and represents the most likely assignments. The abundance of one detected protein was quantified using the average abundance of the top-3 peptides assigned to this protein. LC-MS/MS-based proteomics studies are based on peptides. However, deducing protein identities from a set of identified peptides could be difficult because of sequence redundancy, such as the presence of proteins that have shared peptides. Therefore, in bottom-up proteomics data, there is the challenge of handling redundancy in the matching of peptides to protein hits. These redundant proteins are automatically grouped and are not initially displayed in the search results report. The proteins within a group are ranked according to the number of peptide sequences, the number of PSMs, their protein scores, and the sequence coverage. The top-ranking protein of a group becomes the master protein of that group. The grouping is applied using the Parsimony Principle, protein hits may be reported as the minimum set that accounts for all observable peptides. This may be applied to reduce the protein list where peptides could belong to several proteins (referred by Proteome Discoverer). In metaproteomics studies, the number of unique peptides and also the peptide count per protein is low, so that only one, two, three peptides per protein groups are assigned. All peptides were measured in high resolution mode at the Q Exactive HF instrument which means that the quality and confirmation of identity is highly given. A total of 4,037 protein groups and 5,637 peptides were identified in the metaproteome of 36 soil samples.

The abundance of all detected proteins in each sample was normalized to the abundance of a given protein. The “PROteomics results Pruning & Homology group ANotation Engine” (PROPHANE) (http://www.prophane.de/index.php?p=new) was used to assign proteins to their phylogenetic and functional origin. The abundance of each protein was determined according to the area underneath the curve (AUC) of the corresponding peptides.

We anticipate that fungal proteins were not found among the dominant genera. Three reasons can explain the absence of fungal proteins: (i) the number of fungal genomes is more limited and more importantly, (ii) eukaryotic DNA has introns and, hence, contigs of the same length for bacteria and fungi would translate into shorter peptides for fungi than for bacteria. This complicates the annotation and identification of fungal proteins; and (iii) as mentioned above, pest practices included the utilization of fungicides that could reduce fungal biomass.

### Statistical and data analysis

The normality and variance homogeneity of the variables were tested by the Kolmogorov-Sminorv and Levene tests, respectively. The Shannon-Wiener index of diversity was calculated using the abundance of the active microbial populations at the genus level (active phylogenetic diversity) and the abundance of proteins retrieved from cluster of orthologues groups (COG) at sub role level (functional diversity).

The experimental design included three factors: i) *irrigation quality*, which has two levels: water from the “Tagus-Segura” canal (TW), that does not show high EC or soluble OM; and reclaimed water (RW), that contains high levels of salts and soluble OM derived from the wastewater treatment process; ii) *irrigation quantity*, that also has two levels: the optimal amount of water during the whole year (C); and regulated deficit irrigation (RDI); and iii) *sampling time* (August, January, and June). A three-way ANOVA was used for testing the significance of these factors on the observed variables (dissolved organic C, EC, soil respiration, active diversity and functional diversity). In order to determine pair-wise differences between the treatments at each sampling time, the data were analyzed using one-way ANOVA followed by the Tukey post-hoc test. Differences at *P* < 0.05 were considered as statistically significant. In order to analyze the structure of the microbial community, principal component analyses (PCA) with the relative abundances of proteins at the order level (phylogenetic structure) and COGs (functional structure) were performed. PERMANOVA with 9999 permutations was applied to test the influence of the factors analyzed on the structure of the microbial communities. The statistical analysis was performed using IBM-SPSS Statistics (version 19.0) and R software v.3.1.3.

## Electronic supplementary material

Supplementary Information
