# Supplementary material for: Ecological and functional adaptations to water management in a semiarid agroecosystem: a soil metaproteomics approach
Source: Sci Rep. 2017 Aug 31;7:10221. doi: 10.1038/s41598-017-09973-w (PMC5579227; doi:10.1038/s41598-017-09973-w)
Supplement: Supplementary file 1 — Supplementary Information [file 41598_2017_9973_MOESM1_ESM.pdf]

SUPPLEMENTARY INFORMATION

Ecological and functional adaptations to water management in a semiarid agroecosystem: a soil metaproteomics approach

Robert Starke[1^], Felipe Bastida[2^], Joaquín Abadía[2], Carlos García[2], Emilio Nicolás[2], Nico Jehmlich[1]

[1]Helmholtz-Centre for Environmental Research – UFZ, Department of Molecular Systems Biology, Permoserstrasse 15, 04318 Leipzig, Germany

[2]Centro de Edafología y Biología Aplicada del Segura. Spanish Research Council (CEBAS-CSIC). Campus Universitario de Espinardo, CP 30100 PO Box 164, Murcia, Spain

[^]contributed equally to this study

***Corresponding author: fbastida@cebas.csic.es**

Phone: +34 968396106; Fax: +34 968396213

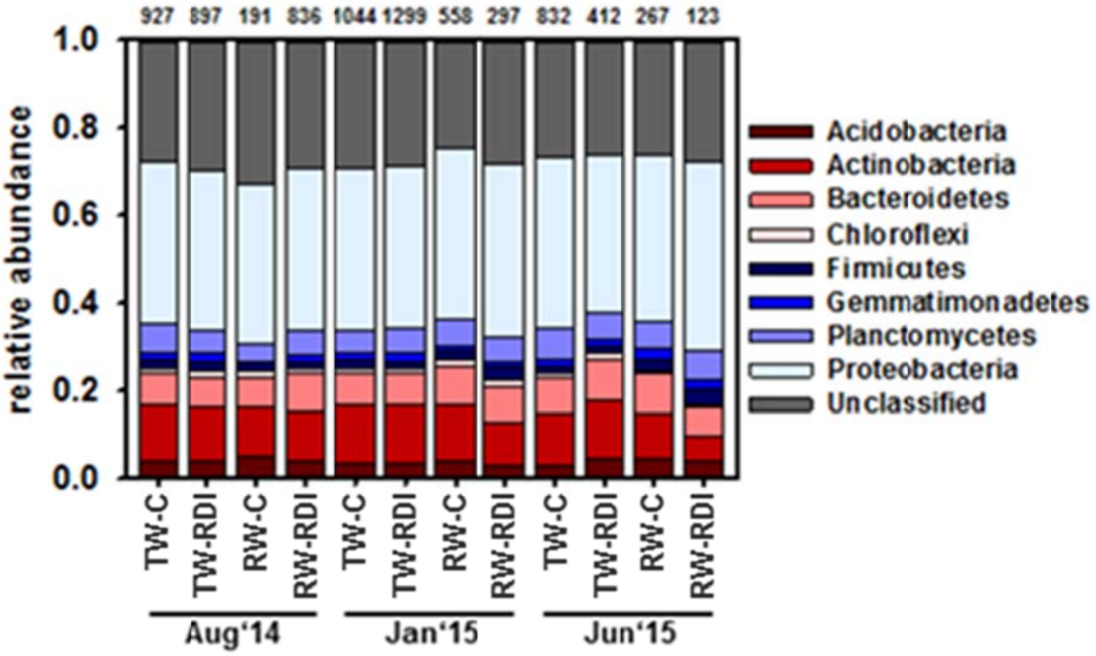

Figure S1 | Relative abundances of the dominant phyla of the soil community. TW-C stands for transfer water control from Tajo River, RW-C for reclaimed water control and RDI for regulated deficit irrigation.

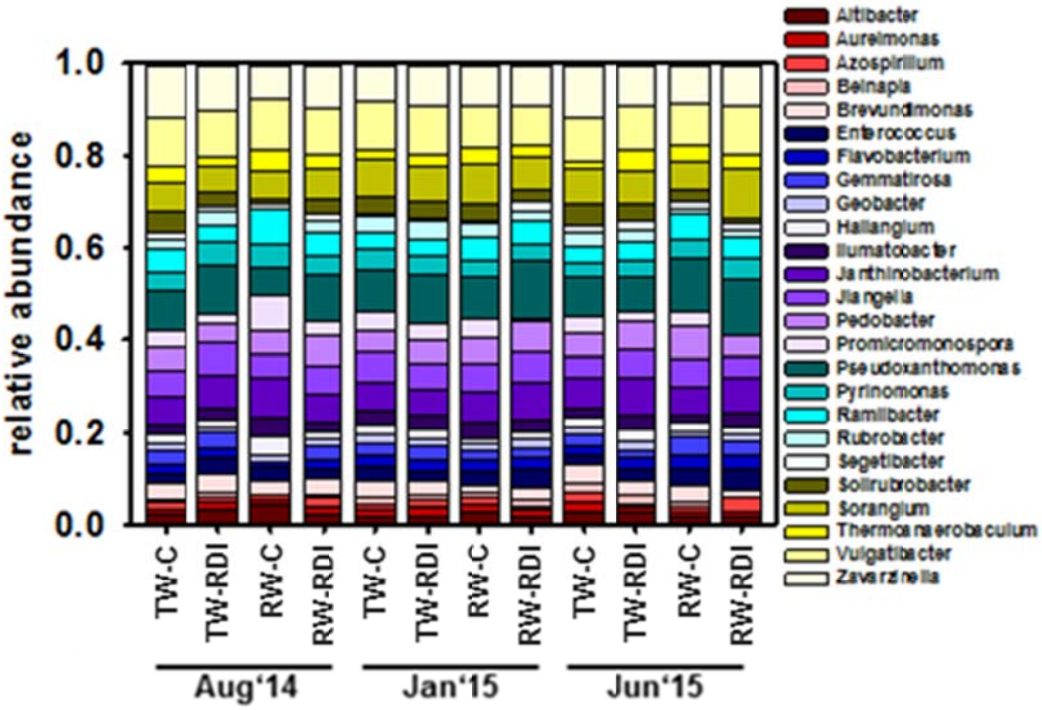

Figure S2 | Relative abundances of the top25 genera of the soil community. TW-C stands for transfer water control from Tajo River, RW-C for reclaimed water control and RDI for regulated deficit irrigation.
